# The enduring power of social context: Pre‐war contact opportunity amplifies the effects of post‐war contact

**DOI:** 10.1111/bjso.70096

**Published:** 2026-05-18

**Authors:** Zaur Afandiyev, Sandra Penić, Sammyh Khan, Sabina Čehajić‐Clancy

**Affiliations:** ^1^ Department of Psychology Stockholm University Stockholm Sweden; ^2^ Department for Political Science and International Relations, and Swiss Center for Affective Sciences University of Geneva Geneve Switzerland; ^3^ School of Behavioural, Social and Legal Sciences Örebro University Örebro Sweden

**Keywords:** contact willingness, intergroup contact, pre‐war contact opportunity, social context

## Abstract

Intergroup contact is one of the most established approaches for improving relations between adversary groups in conflict settings; yet little is known about whether its effects might be shaped by the social context. In this paper, we examine whether pre‐war contact opportunity with the adversary group shapes the relationship between post‐war intergroup contact and willingness to engage with the adversary group, measured at two post‐war time points. We operationalize pre‐war contact opportunity objectively, using pre‐war census data on adversary group share. We analyse data from two studies conducted in post‐war Bosnia and Herzegovina. Study 1, conducted approximately 10 years after the war's outbreak (*N* = 1967), demonstrates that post‐war intergroup contact is more strongly associated with contact willingness among individuals who, before the war, lived in municipalities with higher contact opportunity. Study 2, conducted more than 30 years after the war, does not provide evidence for the same moderating pattern (*N* = 330), but supplementary analyses indicate persistent significant yet attenuated effects of contact opportunity. These findings highlight the relevance of the pre‐conflict social context and temporal dynamics for understanding intergroup contact effects in post‐conflict societies.

## INTRODUCTION

Having positive interactions with members of adversary outgroups in contexts of intergroup conflict is not an easy or frequent endeavour. At the same time, intergroup contact itself is one of the most prominent strategies for improving intergroup relations (Pettigrew & Tropp, 2006), increasing contact willingness (Tang et al., 2024; Yucel & Psaltis, 2020), even in conflict‐affected contexts (e.g. Cehajic et al., 2008; Čehajić‐Clancy et al., 2025). These findings have laid the ground for an expectation that facilitating positive contact between adversary group members contact would be sufficient to disrupt the vicious cycle of contact avoidance and over time facilitate improvement of intergroup relations (Čehajić & Brown, 2010). However, the effects of intergroup contact are more nuanced, particularly in contexts of conflict, than the assumption that a mere contact experience may lead to immediate improvements in intergroup relations (Shulman et al., 2024). In fact, discrete contact experiences, even when positive, are difficult to generalize to the entire outgroup to produce beneficial effects (Paolini et al., 2014), and in some cases, such experiences may even backfire (Paolini et al., 2016).

Existing studies suggest that one important moderator of the effects of contact is *prior* contact experience (Al Ramiah et al., 2013). On the one hand, prior positive contact experience may reduce chronic intergroup anxiety, thereby buffering against anxiety during intergroup interactions (even when experiences are negative) and encouraging contact willingness in the future (Paolini et al., 2016). On the other hand, prior contact may increase category salience during intergroup interactions, which in turn facilitates generalization from the individual contact partner to the broader outgroup (Paolini et al., 2014). In any case, existing evidence suggests that prior contact experience determines the effects of current contact in meaningful ways.

One context in which prior contact may be prevalent and relevant is in ethnically diverse societies that have experienced interethnic conflict. Given that many interethnic conflicts are preceded by a history of ethnic diversity (Montalvo & Reynal‐Querol, 2005; Novta, 2016), post‐conflict societies can indeed be characterized by substantial prior interethnic contact opportunities. This suggests that, although conflict generally hinders willingness to interact with the adversary group, post‐conflict contexts may hold untapped potential—especially among individuals who have had positive contact with outgroup members before the war. We thus review evidence on the relationship between ethnic diversity and positive intergroup contact, focusing in particular on the role of pre‐war ethnic diversity in shaping contemporary intergroup relations.

More specifically in this paper, we examine the moderating effects of pre‐war contact opportunity with members of adversary groups on the relationship between current positive intergroup contact and willingness to interact with the adversary group. We test our predictions in Bosnia and Herzegovina, a post‐war setting with a long pre‐war cohabitation under the Socialist Federal Republic of Yugoslavia. We report findings from two studies: Study 1 reports data collected approximately 10 years after the war's outbreak, and Study 2 (preregistered) reports data collected more than 30 years after the war, allowing us to evaluate the impact of the pre‐war social context over a longer period of time. Across both studies, we estimate a cross‐level interaction between current positive intergroup contact and pre‐war contact opportunity using multilevel models while controlling for relevant individual‐ and context‐level covariates.

### Ethnic diversity and intergroup relations

The effect of ethnic diversity on intergroup relations is neither clear nor straightforward. Meta‐analyses indicate that ethnic diversity generally has a moderately negative impact on social cohesion (Dinesen et al., 2020), primarily as a result of the segregation, absence of intergroup contact and increased perceptions of threat (Laurence & Goebel, 2024; Laurence, Schmid, & Hewstone, 2019). Relatedly, Dixon et al. (2023) describe an ‘*irony of proximity*’, whereby spatial closeness to outgroup members can, depending on the socio‐spatial context, either heighten perceived threat and contact avoidance or facilitate improved intergroup relations through more frequent positive interaction. In addition, studies that have identified this rather negative impact of ethnic diversity on intergroup relations have primarily focused on diversity resulting from recent immigration. Indeed, in such recently diversified contexts, ethnic diversity may not lead to positive intergroup outcomes. On the other hand, when ethnic diversity is not the result of a recent immigration but is instead a long‐standing feature of a social context, ethnic diversity is expected to have more positive effects on intergroup relations (Brunarska & Toruńczyk‐Ruiz, 2023; Gundelach, 2014). In other words, existing evidence indicates that short‐lived diversity tends to have negative effects for intergroup relations, whereas long‐lived diversity may actually exert more positive effects.

These findings align with the notion that initial negative effects of diversity might diminish over time because the contact opportunities created by the very same diversity are utilized to gradually improve intergroup relations (Enos, 2014; Ramos et al., 2019). Considering this, in the contexts where diversity has historically coexisted with high levels of positive intergroup contact, studies show that ethnic diversity indeed elicits beneficial effects on intergroup relations (Hewstone, 2015; Schmid et al., 2014). Furthermore, recent evidence suggests that such benefits of ethnic diversity are not limited to those who personally experience contact: contextual‐level contact (living in settings where positive intergroup encounters are frequent) predicts positive intergroup outcomes over and above individual contact (Čehajić‐Clancy et al., 2025; Christ et al., 2014; Penic et al., 2017).

However, violent political conflicts can profoundly disrupt and reshape intergroup contact experiences by altering the demographic landscape and increasing negative emotions in intergroup encounters. Even where war has disrupted contact, local residents may retain memories of positive interactions that occurred prior to the conflict. Yet, previous research has rarely examined the impact of pre‐war contact opportunity (living in areas with high levels of inter‐ethnic contact opportunity) on current intergroup relations and, in particular, how such past contextual conditions may shape the effects of present‐day intergroup contact in a context of intergroup conflict.

Relevant empirical studies suggest that pre‐war ethnic diversity can exert a transgenerational influence on post‐conflict intergroup relations by shaping the attitudes of individuals who did not directly experience the period of interethnic coexistence. For example, recent studies demonstrate that learning about the historical ethnic diversity of one's place of residence improves intergroup attitudes by enhancing local historical knowledge, which in turn strengthens attachment to the place and fosters more positive views of both current and former residents (Stefaniak et al., 2022). Similarly, studies on transgenerational influences demonstrate that knowledge of ancestral intergroup contact (Stasiuk & Bilewicz, 2013) or exposure to media portrayals of pre‐war intergroup coexistence (Afandiyev & Bilewicz, 2025) can enhance contemporary intergroup attitudes among individuals who did not personally live during that time. These studies suggest that knowledge of ancestral or past contact increased the inclusion of others in the self and facilitated perceived similarity, which in turn improved attitudes towards the outgroup (Stasiuk & Bilewicz, 2013).

In summary, there is converging evidence that learning about past diversity can play a positive role in shaping contemporary intergroup relations (via transgenerational pathways), but there remains a clear empirical gap regarding the effects among individuals who personally and directly experienced ethnic diversity in the past.

### Ethnic diversity as a moderator of intergroup contact effects

Apart from its direct effect on intergroup relations, ethnic diversity—although explored in a relatively limited number of studies—has also been examined as a moderator of the relationship between contact and outgroup attitudes. The direction of the moderating effect remains inconclusive. On the one hand, this moderating effect was introduced as the ‘Wallpaper Effect’ (Barlow et al., 2013), suggesting that a high outgroup share in neighbourhoods may limit the prejudice‐reducing effects of contact for minority group members. However, subsequent studies from multiple contexts did not support this finding, concluding that intergroup contact has similar effects regardless of the level of ethnic diversity (Schmid et al., 2017). Additional support for the absence of ethnic diversity as an inhibitor of contact's prejudice‐reducing effects comes from a recent meta‐analytic study, which revealed that intergroup contact remains effective even under conditions of high outgroup threat and discrimination—features commonly associated with ethnically diverse settings (Van Assche et al., 2023).

As with the direct effect of ethnic diversity on intergroup relations, the moderating effect of diversity on the contact—prejudice relationship is also likely to differ between pre‐war ethnic diversity—typically characterized as long‐standing ethnic diversity—and more recent diversity, such as the one resulting from immigration. Since no studies to date have directly examined whether pre‐war ethnic diversity moderates the relationship between current intergroup contact and contact willingness with the adversary group, in this study, we answer this question by conceptualizing pre‐war ethnic diversity as a proxy for prior intergroup contact, which can shape the impact of subsequent contact experiences. Importantly, we do not assume that contact opportunity necessarily translates into actual intergroup contact. As discussed earlier, a substantial body of research demonstrates that residential proximity or ethnic diversity does not automatically result in interaction and may, under certain conditions, even be associated with avoidance or heightened threat perceptions (e.g. Dixon et al., 2020, 2023; Laurence, Schmid, & Hewstone, 2019; Laurence, Schmid, Rae, & Hewstone, 2019; Paolini et al., 2026). At the same time, existing work suggests that under specific socio‐spatial and institutional conditions, including long‐standing ethnic diversity, lower levels of residential segregation, and normative or institutional support for intergroup mixing, contact opportunity is more likely to translate into actual intergroup contact and, in turn, more positive intergroup relations (Brunarska & Toruńczyk‐Ruiz, 2023; Čehajić‐Clancy et al., 2025; Gundelach, 2014; Laurence, Schmid, Rae, & Hewstone, 2019).

We argue that the pre‐war context of Bosnia and Herzegovina is likely to exemplify such a case in which contact opportunity was widely utilized. Pre‐war Bosnia and Herzegovina was characterized by long‐standing ethnic diversity embedded in everyday social life, and multiple indicators point to a relatively high prevalence of positive intergroup contact. For example, between 1987 and 1989, 11.2% of marriages were exogamous (Botev, 1994), indicating substantial levels of intimate cross‐group contact. Similarly, a 1989 study reported the highest levels of national tolerance in Bosnia and Herzegovina among the Yugoslav republics, plausibly reflecting more frequent and normalized intergroup interaction (Hodson et al., 1994).

### Moderating role of pre‐war contact experiences on post‐war contact effectiveness

Empirical research on the moderating role of pre‐war contact experiences remains limited while yielding mixed findings. For example, Voci et al. (2017) found that post‐war contact had a particularly strong positive effect on intergroup outcomes when pre‐war contact levels were high. In contrast, Kende et al. (2022) suggested that post‐war contact can compensate for the absence of pre‐war contact. In other words, among individuals who established positive intergroup contact after the war, the level of pre‐war contact did not appear to be a determining factor (Kende et al., 2022). Apart from the fact that these studies were conducted in different contexts, characterized by different pre‐war and post‐war circumstances, these discrepancies may, in part, stem from differences in the temporal distance from the conflict. Although data for both studies were collected at approximately the same time (around 2010), the pre‐war contact experiences they examined occurred at very different historical moments. In the Cypriot study (Kende et al., 2022), pre‐war contact referred to experiences that took place approximately 36 years earlier, whereas in the Bosnian study (Voci et al., 2017), pre‐war contact occurred roughly 18 years before data collection. Additionally, a notable limitation across both studies is that pre‐war contact was measured retrospectively, relying on participants' recollection of contact frequency before the conflict. This approach introduces an important bias, as it is plausible that individuals with lower current levels of prejudice may recall pre‐war positive intergroup contact more frequently than it actually was, whereas those with higher prejudice may underreport or selectively forget such contact. In this paper, we address this important limitation by measuring pre‐war contact opportunity using objective indicators such as census data.

Some insight into whether pre‐war contact moderates the effects of post‐war contact comes from studies on how prior contact shapes the subsequent impact of contact. Evidence is mixed, but two models suggest why prior contact could strengthen effects. The contact‐threshold model (MacInnis & Page‐Gould, 2015) proposes that benefits emerge only after cumulative contact surpasses a threshold; below it, single encounters can be null or even harmful. Similarly, the anxiety‐learning model (Paolini et al., 2016) holds that discrete interactions may trigger anxiety, whereas repeated positive contact lowers chronic intergroup anxiety and buffers anxiety in new encounters. Taken together, these accounts imply that prior contact can amplify and may sometimes be necessary for the positive effects of later contact.

## CURRENT RESEARCH

The present work advances existing research by examining the role of pre‐war contact opportunity at two distinct time points following the war within the same post‐conflict context, thereby allowing us to assess how the influence of pre‐war social context on post‐war intergroup contact unfolds over time more directly than prior studies conducted across different conflict settings and historical periods (Kende et al., 2022; Voci et al., 2017). Furthermore, rather than conceptualizing pre‐war contact as retrospectively reported individual experience (Kende et al., 2022; Voci et al., 2017), we reconceptualize it as a structural and historically embedded contact opportunity, operationalized through pre‐war ethnic diversity at the contextual level. This shift allows us to examine how objective exposure to outgroup members in the past might shape the effectiveness of later contact while reducing bias inherent in subjective recall. To address the scarcity and ambiguity in research on how pre‐war ethnic diversity shapes contact effectiveness, we conducted two studies examining whether pre‐war contact opportunity with members of adversary groups moderates the relationship between post‐war intergroup contact and willingness to engage with the adversary group. Specifically, we use pre‐war ethnic diversity—operationalized as the level of contact opportunity in a respondent's pre‐conflict environment—as a contextual and objective indicator.

### Study 1

To test our hypothesis, we conducted a study in Bosnia and Herzegovina. This context is well suited to our questions: prior to the 1992–1995 war, Bosnia and Herzegovina was part of the Socialist Federal Republic of Yugoslavia, where Bosniaks, Serbs, and other ethnic groups lived under shared state institutions for nearly half a century (Malcolm, 1996). This long‐standing cohabitation provided municipality‐level variation in pre‐war contact opportunity, offering a suitable context in which to test our hypothesis.

#### Sample

In order to test our hypothesis, we used data collected within the Southeast European Social Survey Programme, conducted in late 2003 and early 2004 (Simkus, 2007). All participants provided informed consent. Out of the total sample size of 18,902, 6809 respondents were from Bosnia and Herzegovina, of whom 4654 identified as either ethnic Bosniaks or Serbs. In this study, we focus only on Bosniaks and Serbs as relations between these two ethnic groups can be regarded as clearly adversarial. Since the survey did not include any questions about the place of residence before the war, we excluded from the analysis all individuals who replied ‘No’ to the question, ‘Are you living in the same republic, and region of the republic, in which you were living when you were 14 years old?’ Additionally, we excluded those who did not answer the questions about intergroup friendship and contact willingness with the adversarial group. After applying these exclusion criteria, our final sample consisted of 1967 respondents from 105 (out of 142) different municipalities in Bosnia and Herzegovina.

#### Measures

##### Individual‐level indicators

*Intergroup contact* was measured with a single question: ‘Thinking now about your friends, say your ten closest friends, are any of these friends of a different nationality than you?’ Respondents were given ‘Yes’ or ‘No’ response options, making this variable dichotomous.

*Contact willingness with the adversary group* was measured using a modified version of Bogardus's (1925) social distance scale with 8 questions (e.g. ‘Would you like a person of Serb/Bosnian Muslim ethnic group living permanently in your country?’; ‘Would you like a person of Serb/Bosnian Muslim ethnic group as your friend or companion to spend time with?’). Responses were recorded on a 3‐point scale (1 = It makes no difference to me, 3 = I would really have strong objections). Respondents answered these questions for 20 different ethnic groups. Internal consistency was acceptable for responses from Bosniaks about Serbs and from Serbs about Bosniaks (Cronbach's *α* = .90 and .93, respectively). The average score for the questions given by Bosniaks about Serbs and by Serbs about Bosnian Muslims, was used as the measure of adversary social distance. This score was then reverse‐coded so that higher values indicated greater willingness for contact with the adversary group.

###### Individual‐level control variables

We adjusted for covariates that could potentially confound the predicted moderation effect. Controls included basic demographics—*age, sex*(Male(1)/Female(0)), *ethnic group* (Bosniaks(1)/Serbs(0)) and *war experience* linked to lower willingness for contact with the adversary (Kauff et al., 2021) and measured with a single question: ‘Have you experienced or witnessed a war‐related event that involved actual or threatened death or injury to which you responded with intense fear, helplessness, or horror?’ Respondents answered with ‘Yes’(1) or ‘No’(0).

##### Context‐level indicators

*Pre‐war contact opportunity* was measured as adversary share based on the population census of Yugoslavia conducted just before the start of the war, in 1991. For each of the 106 municipalities, our data includes two contact opportunity values: for Bosniaks, this represents the share of Serbs in the municipality and for Serbs, it represents the share of Bosniaks.

###### Context‐level control variables

*Type of settlement* (Rural(1)/Urban(0)) and adversary share increase were used as municipality‐level control variables. *Adversary share increase* was measured based on the population census of Yugoslavia conducted in 1991 and the most recent available census data closest to the time of the survey, which was conducted in Bosnia and Herzegovina in 2013. As with the measure of contact opportunity before the war, for each municipality we have two adversary share increase values: for Bosniaks, this represents the change in the share of Serbs in the municipality between the 1991 and 2013 censuses; for Serbs, it represents the change in the share of Bosniaks in the municipality over the same period. Additionally, we controlled for historical educational attainment at the municipal level to address potential alternative explanations, as areas with higher pre‐war contact opportunity may also differ systematically in educational composition. Given that higher education is consistently associated with outgroup contact willingness (Hello et al., 2006), we included pre‐war municipal educational attainment based on the 1991 Yugoslav census, measured as the share of the population with higher education.

### Results

Descriptive statistics and bivariate correlations between the variables are presented in Table 1. The mean level of contact willingness with the adversary group was 2.08 (SD = 0.66), indicating a moderately high willingness on a 3‐point scale. Men reported slightly lower willingness than women (*r* = −.06, *p* < .01), as did respondents with war experience (*r* = −.06, *p* < .01), whereas age and settlement type were unrelated to the outcome. Bosniak respondents reported higher willingness than Serb respondents (*r* = .34, *p* < .01). Intergroup friendship was strongly positively associated with contact willingness (*r* = .44, *p* < .01) and adversary share increase was also positively associated with willingness (*r* = .17, *p* < .01). In contrast, pre‐war contact opportunity was not significantly correlated with contact willingness (*r* = −.00).

**TABLE 1:** Descriptive statistics and bivariate correlations (Study 1).

Variable	*M*	*SD*	1	2	3	4	5	6	7	8	9
1. Intergroup friendship	0.54	0.50									
2. Pre‐war contact opportunity	0.22	0.16	.04								
3. Contact willingness with adversary group	2.10	0.66	.44**	−.01							
4. Age	40.45	15.94	.01	−.01	−.00						
5. Sex	0.47	0.50	.02	−.01	−.05**	−.03					
6. Ethnic group	0.58	0.49	.29**	−.29**	.34**	.02	−.11**				
7. War experience	0.51	0.50	−.13**	−.00	−.08**	−.08**	−.16**	−.17**			
8. Type of settlement	0.56	0.50	.17**	.02	.08**	.02	.00	.07**	−.02		
9. Adversary share increase	−0.09	0.13	.12**	.06**	.17**	.07*	.00	−.09**	.03	−.01	
10. Pre‐war municipal educational attainment	0.04	0.03	.15**	.25**	.04	−.05	−.02	.07**	−.04	.09**	.04*

*Note*: *M* and *SD* are used to represent mean and standard deviation, respectively. * indicates *p* < .05. ** indicates *p* < .01.

To test our hypothesis, we conducted Bayesian multilevel analyses using the *brms* package in R. Models were estimated using Hamiltonian Monte Carlo with the No‐U‐Turn Sampler. To robustly assess the interaction between pre‐war contact opportunity and post‐war intergroup contact, we included a random slope for intergroup contact (Heisig & Schaeffer, 2019). All Bayesian models were estimated using four independent Markov chain Monte Carlo (MCMC) chains, each run for 4000 iterations, with the first 2000 iterations discarded as warmup, yielding 8000 post‐warmup draws per model. Model convergence and estimation quality were assessed using visual inspection of trace plots, effective sample sizes, and the potential scale reduction factor (R‐hat). Across all fixed and random effects, R‐hat values were below 1.01, effective sample sizes were sufficiently large and trace plots indicated good mixing and no evidence of non‐stationarity, supporting reliable posterior inference.

Intergroup contact was decomposed into a group‐mean–centred (within‐municipality) component and a municipality‐level (between‐municipality) component. Interactions between each component and pre‐war contact opportunity were estimated in order to correctly identify the level at which the interaction occurs and to avoid conflating within‐ and between‐context effects (Enders & Tofighi, 2007). All predictors expressed as shares (pre‐war contact opportunity, adversary share increase and pre‐war municipal educational attainment) were standardized prior to analysis, such that estimated effects correspond to a one‐standard deviation change rather than a one‐unit (i.e. 100‐percentage‐point) change, facilitating substantively meaningful interpretation of coefficients.

**FIGURE 1:**
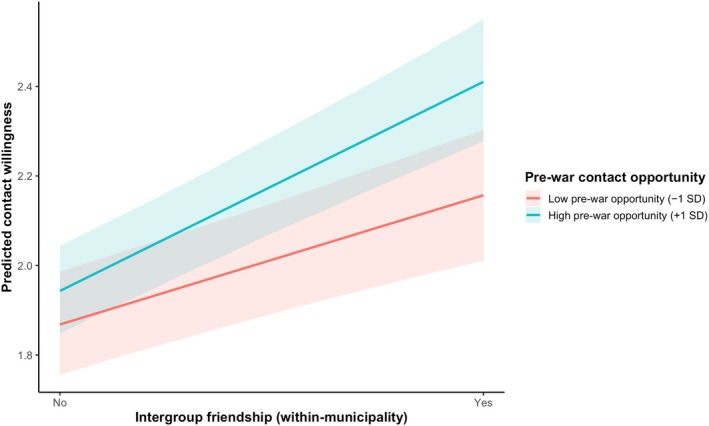
The enduring impact of social context 10 years after the war: Moderating effect of objective pre‐war contact opportunity on the relationship between positive post‐war intergroup friendship and contact willingness with the adversary group (Study 1). Lines show predicted contact willingness at two levels of objective pre‐war contact opportunity (low = −1 SD; high = +1 SD). The x‐axis indicates current positive intergroup friendship (within‐municipality; ‘No’ vs. ‘Yes’). Predictions are estimated at the mean level of municipality‐level contact. Shaded areas represent 95% credible intervals. Analyses are based on 1967 respondents (Level 1) nested within 105 municipalities (Level 2).

Results of the estimated Bayesian multilevel model are presented in Table 2. At the individual (within‐municipality) level, intergroup friendship was positively associated with contact willingness (B = 0.38, 95% CI [0.31, 0.45]). At the between‐municipality level, the average level of intergroup friendship within the municipality was also positively associated with contact willingness (B = 1.11, 95% CI [0.77, 1.45]). Pre‐war contact opportunity itself was not directly associated with contact willingness (B = 0.01, 95% CI [−0.10, 0.13]). However, consistent with our central hypothesis, pre‐war contact opportunity moderated the association between individual‐level intergroup friendship and contact willingness. The interaction between within‐municipality intergroup friendship and pre‐war contact opportunity was positive and robust (B = 0.09, 95% CI [0.03, 0.15]), indicating that the positive association between intergroup friendship and contact willingness is stronger in municipalities where opportunities for intergroup contact were greater prior to the war. This pattern is illustrated in Figure 1, which presents within‐municipality contrasts between individuals who do and do not report an intergroup friend while holding the municipality‐level average of intergroup friendship constant at the grand mean. As shown in the figure, intergroup friendship is positively associated with contact willingness across all levels of pre‐war contact opportunity, but the magnitude of this association increases substantially as prior contact opportunity rises. In contrast, the corresponding interaction at the between‐municipality level was not credibly different from zero (B = 0.05, 95% CI [−0.14, 0.23]), suggesting that the moderating role of pre‐war contact opportunity operates primarily at the individual level rather than through aggregated municipality friendship levels. We conducted additional robustness analyses, which yielded substantively similar results (see Appendix S1).

**TABLE 2:** Bayesian multilevel linear regression predicting high contact willingness with the adversary group (Study 1).

Term	Estimate	95% CI
Intercept	1.44	[1.23, 1.64]
Within level friendship	**0.38**	**[0.31, 0.45]**
Between level friendship	**1.11**	**[0.77, 1.45]**
Pre‐war contact opportunity	0.01	[−0.10, 0.13]
Within level friendship × Pre‐war contact opportunity	**0.09**	**[0.03, 0.15]**
Between level friendship × Pre‐war contact opportunity	0.05	[−0.14, 0.23]
Age	−0.00	[−0.00, −0.00]
Sex	−0.02	[−0.07, 0.02]
Ethnic group	**0.12**	**[0.02, 0.21]**
War Experience	0.02	[−0.03, 0.07]
Type of settlement	0.01	[−0.03, 0.06]
Adversary share increase	**0.09**	**[0.05, 0.12]**
Pre‐war municipal educational attainment	−0.05	[−0.12, 0.02]
Sample size	1967	
Number of municipalities	105	
ICC (null model)	.44	

*Note*: Bold values indicate 95% credible intervals. Effects were interpreted as credibly different from zero when the 95% credible interval excluded zero.

### Discussion

The results of this study provide support for our hypothesis. Pre‐war contact opportunity, as an indicator of a pre‐war social context, played an important role in post‐war intergroup relations. More specifically, 10 years after the war, current intergroup friendships were more strongly associated with contact willingness among respondents who, prior to the war, lived in municipalities with higher intergroup contact opportunities. Importantly, this moderation effect was cross‐level: when comparing two individuals living in the same municipality, one who had an intergroup friend and one who did not, the difference in their contact willingness is larger in municipalities that had higher pre‐war contact opportunity. By contrast, when comparing municipalities with lower versus higher average levels of intergroup friendship, differences in average contact willingness between municipalities do not depend on whether those municipalities had higher or lower pre‐war contact opportunities.

As stated above, we operationalized pre‐war contact opportunity using the pre‐war census share of the adversary group in the respondent's municipality. This objective, contextual measure mitigates concerns about attitudinal self‐report bias (e.g. that respondents with more favourable outgroup attitudes would retrospectively overestimate pre‐war opportunity), thereby strengthening the credibility of the moderation effect. Importantly, this moderation effect remained significant even after controlling for relevant individual‐level covariates (e.g. age, sex, ethnic group, war experience) and context‐level covariates (e.g. adversary share increase, type of settlement, pre‐war municipal educational attainment), further demonstrating the robustness of the findings.

Taken together, this study demonstrates the impact of pre‐war contact opportunity on amplifying the effects of intergroup contact approximately 10 years after the war, in a manner similar to the findings of (Voci et al., 2017), set in the same context 18 years after the war. However, the durability of this moderating effect over a longer time remains uncertain. As noted above, another study addressing a similar research question, although in a different conflict context (Kende et al., 2022), after a longer time gap (36 years after the war), did not identify any moderating effect of pre‐war contact experience on the impact of post‐war contact. Our second study therefore primarily examines the durability of the moderating role of pre‐war contact opportunity by collecting data 30 years after the war.

### Study 2

To examine the durability of the moderating role of pre‐war contact opportunity, we conducted an additional preregistered study (osf.io/8f2mk). In the preregistration, we specified that—consistent with Study 1—the effect of current positive intergroup contact on willingness to interact with the adversary group would be stronger among respondents who had lived before the war in municipalities with higher contact opportunity with the adversary group. Data was analysed using the same strategy as in Study 1. However, the two studies differed in how intergroup contact was measured: Whereas Study 1 used a dichotomous indicator of whether the respondent had at least one intergroup friendship, Study 2 measured positive contact specifically with the adversary group on a 7‐point Likert scale, providing greater variation and a clear outgroup specification. Furthermore, the studies differ in timing in relation to when the war ended: Study 1 analysed data collected roughly 10 years after the onset of the war, whereas Study 2 examines outcomes more than 30 years later, allowing an assessment of the enduring effects of pre‐war contact opportunity.

#### Sample

Data that was used for this study constituted a part of another large‐scale experimental study, which included four experimental groups and one control group (total *N* = 7200). Informed consent was obtained from all participants. For this study, we focused on data obtained from the control group. The study employed a stratified, clustered random sampling design, with municipalities as primary sampling units and polling areas or rural settlements as secondary sampling units, yielding a sample representative of the 18‐ to 65‐year‐old Bosniak and Serb population in Bosnia and Herzegovina. Randomization to study condition was implemented at the level of the primary sampling unit (polling area or settlement), such that all respondents within a given unit were assigned to the same condition. This cluster‐level randomization design eliminated the possibility of spillover effects between treatment and control participants. In order to test our hypothesis, we used data from the control group of the first wave of a multi‐wave study. The control group comprised 2400 respondents, evenly split between self‐identified Bosniaks (*N* = 1199) and Serbs (*N* = 1201). Surveys were administered across 100 municipalities, with 24 respondents per municipality. Because the analyses are restricted to control‐group respondents, the reported effects cannot be attributed to experimental manipulation.

For the final analysis (following the pre‐registered selection criteria), we included only respondents who were at least 44 years old, ensuring they had been at least 10 years old at the outbreak of the war in 1992. This age threshold ensured participants could construe past encounters as intergroup (Quintana, 1998) and recall durable memories of pre‐war life (Bauer, 2015). This reduced the sample to 1127 respondents. Since we used pre‐war census data for the respondents' current municipality as an objective measure of pre‐war contact opportunities, the sample was restricted to individuals who lived in the same municipality before the war. Otherwise, the measure would not capture respondents' actual pre‐war experience, as respondents who relocated are linked to pre‐war data from a different municipality. Although the preregistration specified this exclusion, the survey did not include a direct question about residential continuity. As a proxy, we used the question on place of birth, retaining only those whose municipality of birth matched their current municipality. After applying this criterion, the final sample consisted of 330 respondents from 59 municipalities.

#### Measures

##### Individual‐level indicators

*Positive intergroup contact* was measured with two items: (1) Frequency of positive interactions with Serbs/Bosniaks known personally; (2) Frequency of positive interactions with Serbs/Bosniaks not known personally. A 7‐point scale (1 = Never, 7 = Often) was used and responses on two items were averaged. Internal consistency was acceptable (Cronbach's *α* = .83).

*Willingness for intergroup contact* was assessed with three items asking respondents how willing they were to have Serbs/Bosniaks as (1) neighbours, (2) close friends and (3) family members. Items were rated on the same 7‐point scale (1 = Strongly Disagree, 7 = Strongly Agree) and averaged. Internal consistency was acceptable (Cronbach's *α* (Bosniaks about Serbs) = .80; Cronbach's *α* (Serbs about Bosniaks) = .79).

##### Context‐level indicators

*Pre‐war contact opportunity* was also measured as in Study 1, using adversary group share from the 1991 Yugoslav census. For each municipality, this represented the proportion of Serbs for Bosniak respondents and the proportion of Bosniaks for Serb respondents.

##### Control variables

We included the same individual‐level controls as in Study 1: *age*, *sex* (Male = 1, Female = 0), *ethnic group* (Bosniak = 1, Serb = 0), and *war experience*. War experience was coded 1 if respondents reported either (a) the death of an immediate family member during the war (e.g. son, daughter, parent, sibling, grandparent or grandchild) or (b) having been personally wounded; otherwise, it was coded 0. At the municipality level, the *adversary share increase* was measured as in Study 1. We also controlled for *pre‐war municipal educational attainment* (share with higher education in 1991) as in Study 1.

### Results

Table 3 presents descriptive statistics and bivariate correlations. Willingness to interact with the adversary group averaged 4.75 (SD = 1.73) on a 1–7 scale, indicating moderate to high willingness overall. Replicating prior work, positive intergroup contact was strongly associated with greater willingness to interact (*r* = .46, *p* < .001). Willingness showed a small positive association with adversary share increase (*r* = .13, *p* < .05) and with ethnic group membership (*r* = .15, *p* < .01), indicating higher willingness among Bosniaks than Serbs. As in Study 1, willingness was not correlated with objective pre‐war contact opportunity. War experience, age, sex and pre‐war municipal educational attainment were unrelated to the outcome.

**TABLE 3:** Descriptive statistics and bivariate correlations (Study 2).

Variable	*M*	*SD*	1	2	3	4	5	6	7	8
1. Positive intergroup contact	3.85	1.58								
2. Pre‐war contact opportunity	0.25	0.17	.22**							
3. Contact willingness with adversary group	4.75	1.73	.46**	.06						
4. Age	56.10	6.72	.05	.05	.10					
5. Sex	0.49	0.50	−.02	−.05	−.03	.08				
6. Ethnic group	0.56	0.50	−.07	−.26**	.15**	.01	.11			
7. War experience	0.45	0.50	.01	.02	.03	.07	.22**	.21**		
8. Adversary share increase	−0.12	0.16	.07	−.33**	.13*	−.04	−.01	.16**	.16**	
9. Pre‐war municipal educational attainment	0.06	0.04	.08	−.01	.02	−.03	0.6	.08	.07	−.11

*Note*: *M* and *SD* are used to represent mean and standard deviation, respectively. * indicates *p* < .05. ** indicates *p* < .01.

To assess the moderating effect of pre‐war contact opportunity, Bayesian models in Study 2 were estimated using the same multilevel specification and moderation approach as in Study 1, including identical decomposition of intergroup contact into within‐ and between‐municipality components and the same MCMC settings and convergence diagnostics. Results from the multilevel Gaussian model are presented in Table 4. At both the individual and municipality levels, positive intergroup contact was positively associated with willingness to interact (within‐municipality: B = 0.47, 95% CI [0.30, 0.61]; between‐municipality: B = 0.54, 95% CI [0.26, 0.82]). Objective pre‐war contact opportunity showed no direct association with willingness. Neither the within‐ nor between‐municipality interaction between positive intergroup contact and pre‐war contact opportunity was credibly different from zero, providing no evidence for moderation when willingness was modelled continuously. Thus, in contrast to Study 1, there was no evidence that individuals who had lived before the war in more diverse municipalities benefited more strongly from their own current intergroup contact three decades after the war.

**TABLE 4:** Bayesian multilevel linear regression predicting high contact willingness with the adversary group (Study 2—objective pre‐war contact opportunity).

Term	Estimate	95% CI
Intercept	1.49	[−0.30, 3.31]
Within level positive intergroup contact	**0.47**	**[0.30, 0.61]**
Between level positive intergroup contact	**0.54**	**[0.26, 0.82]**
Objective pre‐war contact opportunity	−0.23	[−1.26, 0.82]
Within level positive intergroup contact × Objective pre‐war contact opportunity	−0.00	[−0.17, 0.16]
Between level positive intergroup contact × Objective pre‐war contact opportunity	0.06	[−0.18, 0.30]
Age	0.02	[−0.01, 0.04]
Sex	−0.13	[−0.47, 0.22]
Ethnic group	**0.55**	**[0.07, 1.02]**
War Experience	−0.02	[−0.38, 0.33]
Adversary share increase	0.10	[−0.12, 0.33]
Pre‐war municipal educational attainment	−0.08	[−0.31, 0.14]
Sample size	330	
Number of municipalities	59	
ICC (null model)	.20	

*Note*: Bold values indicate 95% credible intervals. Effects were interpreted as credibly different from zero when the 95% credible interval excluded zero.

Although the continuous Gaussian model was treated as the primary specification, we additionally estimated a dichotomized version of willingness to follow the preregistered analysis plan, which included this specification as a precaution against possible violations of Gaussian assumptions. However, as shown by the posterior predictive checks reported in Appendix S2, the continuous Gaussian model provided an acceptable fit to the observed data. We therefore treat the dichotomized analyses as secondary specification checks rather than as the primary basis for inference. In this preregistered dichotomized specification, some evidence of moderation emerged at the municipality level: pre‐war contact opportunity amplified the association between municipality‐level positive intergroup contact and willingness to interact (OR = 1.95, 95% CI [1.04, 3.95]; Appendix S2, see Appendix S3 for visualization). However, the moderation at the between‐level was not robust across alternative specifications, including changes in the threshold used for dichotomization. The same overall pattern was observed across the additional analyses reported in Appendix S4. Accordingly, unlike in Study 1, Study 2 does not provide evidence that pre‐war contact opportunity amplified the individual‐level association between current positive intergroup contact and willingness to engage with the adversary group, although there are some indications of moderation in the effect of between‐level contact on willingness for contact.

### Discussion

In contrast to Study 1, Study 2 did not provide evidence for moderation by pre‐war contact opportunity when willingness was modelled as a continuous outcome. Although preregistered analyses using a dichotomized outcome yielded some indications of moderation, these effects emerged only at the between‐municipality level, were weaker and less consistent than those observed in Study 1 and depended on model specification. To assess whether this attenuation reflected limited statistical power rather than the absence of moderation, we conducted a series of additional analyses that substantially expanded the analytic sample. These included re‐estimating the models using a larger sample that incorporated participants from both the control and experimental conditions, as well as analyses using perceived pre‐war contact opportunity, which allowed inclusion regardless of post‐war residential mobility. Together, these approaches yielded sample sizes and clustering structures comparable to those in Study 1 and provided sufficient power to detect interactions of a magnitude similar to that previously observed. Even under these expanded specifications, however, the substantive pattern of results remained unchanged: moderation was detected for the effect of between‐municipality‐level contact when contact willingness was dichotomized, while there was no evidence that the within‐municipality‐level contact effect was moderated by pre‐war contact opportunity, as in Study 1. Taken together, these findings suggest that moderation by pre‐war contact opportunity, 30 years later, is present yet attenuated and appears to operate at the between‐municipality level rather than the individual level.

## GENERAL DISCUSSION

Across two studies conducted in the post‐war context of Bosnia and Herzegovina, we find that pre‐war contact opportunity with the adversary group moderates the association between post‐war intergroup contact and willingness to engage with the adversary group in Study 1, with this individual‐level moderating effect no longer evident in Study 2. In Study 1, conducted in the earlier post‐war period, pre‐war contact opportunity significantly amplified the relationship between post‐war contact and contact willingness. In Study 2, conducted approximately three decades after the war, this moderating effect was not observed. The only indications of moderation in Study 2 emerged in dichotomized models and at the between‐municipality level, suggesting a contextual pattern rather than a replication of the individual‐level effect observed in Study 1. Importantly, both studies relied on the same objective, contextual indicator of pre‐war contact opportunity, making recall bias an unlikely explanation for the observed divergence. Together, these findings suggest that the pre‐war social context (operationalized as contact opportunity) shapes the effectiveness of post‐war contact primarily during earlier phases of post‐conflict recovery.

Two theoretical approaches, the contact threshold model (MacInnis & Page‐Gould, 2015) and the habit‐rupture model of intergroup contact (Paolini et al., 2026), might offer plausible explanations for this pattern. From a contact threshold perspective, pre‐war contact opportunity in Bosnia and Herzegovina likely enabled individuals in high‐opportunity municipalities to accumulate sufficient intergroup contact prior to war to reach a point at which contact became reliably beneficial and self‐reinforcing. Crucially, this model assumes a *savings* process: once the threshold is reached, the benefits of accumulated contact are not eliminated by disruption, but can be recovered more rapidly when contact resumes. The war itself constituted a severe interruption to contact accumulation, temporarily halting further exposure without necessarily erasing prior gains. In the earlier post‐war period captured by Study 1, individuals from high pre‐war contact opportunity contexts may therefore have required less renewed contact experience to (re)reach the threshold once opportunities for contact returned, allowing current contact to more readily exert beneficial effects on willingness to engage with the adversary group. By contrast, over the much longer post‐war period captured by Study 2, individuals from low pre‐war opportunity contexts may also have had sufficient time to accumulate post‐war contact and reach the threshold, thereby reducing between‐context differences in the individual‐level moderating role of pre‐war contact opportunity. To provide some additional, albeit indirect, support for this interpretation, we also compared descriptive patterns of intergroup friendship at the two post‐war time points. In Study 1, 46% of respondents reported not having a close friend from another ethnic group. In Study 2, based on the friendship item used in the supplementary analysis, only 15% of the final analytic sample and 18% of the full control‐group sample (*N* = 2400) reported having no friends from the adversary group. While these comparisons should be interpreted cautiously given differences in contact measurement across studies, they are consistent with more widespread intergroup friendship by the time captured in Study 2.

A complementary explanation might also be offered by the habit‐rupture model. In this framework, repeated pre‐war intergroup exposure in high‐opportunity municipalities may have supported the formation of relatively automatic contact routines anchored in stable environmental cues. The war represents an extreme macro‐level rupture that disrupted these cue–response contingencies and produced a period of contact discontinuity. Importantly, research on habit learning suggests that such disruptions do not eliminate earlier learning; rather, previously acquired response tendencies can re‐emerge when contexts again support them (Wood & Rünger, 2016). In the earlier post‐war period, individuals whose pre‐war environments had supported contact habit formation may therefore have been more likely to re‐establish contact routines once opportunities reappeared, amplifying the effects of current contact. Over time, however, as post‐war contexts stabilized and contact opportunities accumulated more broadly, contact habits may have (re)formed even among individuals with limited pre‐war experience, reducing individual‐level differences linked to pre‐war contact opportunity.

These interpretations help situate our findings in relation to previous research. The moderation effect observed in Study 1 closely resembles the pattern reported by Voci et al. (2017), who examined the same conflict context in a relatively early post‐war phase. Both studies likely captured a period in which intergroup contact was still being re‐established following wartime disruption. Under both the contact threshold and habit‐rupture accounts, this period represents a window during which individuals with richer pre‐war contact histories would be advantaged in recovering the benefits of contact, resulting in stronger effects of current contact.

Attenuation of the moderating effect in Study 2 also parallels findings reported by Kende et al. (2022) in the Cypriot context, but the mechanisms underlying this convergence are likely to be different. Although that study was conducted more than three decades after the outbreak of the conflict, meaningful opportunities for intergroup contact were largely absent until the opening of checkpoints in 2003 (Demetriou, 2007). As a result, the effective period of renewed contact accumulation at the time of data collection in 2010 was relatively short—approximately 7 years—comparable in length to the post‐war period captured by our Study 1. From a contact threshold perspective, this would lead to the expectation that individuals who had previously reached the threshold through pre‐war contact experience would again be advantaged in recovering contact benefits. However, no such moderating effect was observed.

This discrepancy might be more comprehensively explained by the habit‐rupture model when combined with principles of contextual renewal from the psychology of habit. Renewal of previously acquired responses is strongest when the post‐disruption context closely resembles the original acquisition context (ABA renewal) and substantially weaker when behaviour is tested in a context that differs from both the acquisition and disruption contexts (ABC renewal) or when the disruption context persists (AB) (Bouton et al., 2011). In Bosnia and Herzegovina, although the war constituted a profound rupture, the post‐war environment did not impose rigid physical separation between groups and many communities retained some degree of residential and social diversity. As a result, post‐war contact opportunities likely reinstated at least some of the environmental cues associated with pre‐war contact, making the Bosnian context more closely approximate an ABA structure. Under such conditions, renewal of pre‐war contact routines would be more likely, allowing pre‐war contact opportunity to amplify the effects of renewed contact—particularly in the earlier post‐war period.

Taken together, the two theoretical models help explain why pre‐war contact opportunity moderated the within‐municipality effect of current contact in the earlier post‐war period, but not three decades after the war. By contrast, the moderation observed in Study 2 at the between‐municipality level may point to a different and even more enduring role of context, although this finding was not highly robust across specifications and should therefore be interpreted cautiously. Because the contextual contact effect is reflected in the difference between the between‐municipality and within‐municipality components of contact, the moderation of the between‐municipality effect in the absence of moderation of the within‐municipality effect suggests that pre‐war contact opportunity may strengthen the contextual component of contact. Given evidence that contextual contact effects operate through norms (Christ et al., 2014), one possible interpretation is that municipalities with historically higher levels of intergroup interaction had developed stronger norms of coexistence before the war, and that the re‐establishment of such norms after the war made contextual contact more consequential for willingness to engage with the adversary group. To our knowledge, no prior research has directly tested whether re‐established norms exert stronger effects than newly emerging ones, so this account remains speculative and should be viewed only as a preliminary interpretation of the observed pattern. Because the between‐municipality moderation effect was not highly robust across specifications, further research is needed to determine whether it replicates reliably and to investigate more thoroughly the mechanisms that may underlie it.

It should be noted as a limitation that, in Study 1, current intergroup contact was operationalized as having a close friend from any other ethnic group, rather than specifically from the adversary group. Consequently, the observed moderating effect of pre‐war contact opportunity in Study 1 cannot be interpreted as moderating only primary transfer effects. Because the contact measure was broader, this moderation may also reflect secondary transfer processes, whereby friendship with a non‐adversary outgroup generalizes to willingness toward the adversary group, as well as broader deprovincialization processes, that is, a less ethnocentric and more open orientation toward other groups (Boin et al., 2021). At the same time, although there is no clear systematic evidence directly comparing the relative strength of these transfer processes, theoretical accounts would generally suggest that primary transfer should have the strongest effects on intergroup outcomes, whereas secondary and tertiary transfer effects are likely to be weaker because they rely on more indirect generalization processes (Meleady et al., 2019). Therefore, if measurement specificity were the key issue, the more specific adversary‐group contact measure used in Study 2 should have yielded an equal or stronger moderation effect, yet this was not observed. This is especially plausible here because pre‐war contact opportunity was measured specifically as an opportunity for contact with the adversary group, rather than with outgroups in general. Additionally, even if the broader contact measure in Study 1 captured not only primary but also secondary or tertiary transfer, it still reflected an intergroup contact effect and therefore does not diminish the central finding that pre‐war contact opportunity amplified contact effects.

Nevertheless, an important implication of these findings for practitioners and policymakers is that individuals who had more extensive everyday *positive* intergroup experience prior to the war may require less intensive intervention to benefit from post‐conflict contact initiatives—particularly in contexts that reinstate features of the pre‐war social environment. At the same time, it is important to note that our studies, as well as the related studies by Voci et al. (2017) and Kende et al. (2022), are correlational in nature, and stronger causal evidence is therefore needed. While experimental manipulation of pre‐war intergroup experience is not feasible, future research could randomize current direct‐contact interventions and examine pre‐war intergroup experience as a measured moderator (Čehajić‐Clancy & Halperin, 2024). In addition, future studies could assess whether pre‐war intergroup experience similarly moderates the effectiveness of other established reconciliation interventions—such as vicarious contact, social‐norms approaches, or moral exemplar interventions—particularly as a function of the extent to which post‐conflict contexts reinstate conditions conducive to contact renewal (Čehajić‐Clancy & Halperin, 2024).

## AUTHOR CONTRIBUTIONS

**Zaur Afandiyev:** Conceptualization; methodology; formal analysis; visualization; writing – original draft; writing – review and editing. **Sandra Penić:** Data curation; conceptualization; writing – review and editing. **Sammyh Khan:** Conceptualization; writing – review and editing. **Sabina Čehajić‐Clancy:** Conceptualization; methodology; project administration; validation; supervision; funding acquisition; writing – original draft; writing – review and editing.

## CONFLICT OF INTEREST STATEMENT

The authors declare no competing interests.

## Supporting information


Appendix S1:

## Data Availability

The data that support the findings of this study are available on request from the corresponding author. The data are not publicly available due to privacy or ethical restrictions.
