# Supplementary material for: The enduring power of social context: Pre‐war contact opportunity amplifies the effects of post‐war contact
Source: Br J Soc Psychol. 2026 May 18;65:e70096. doi: 10.1111/bjso.70096 (PMC13184366; doi:10.1111/bjso.70096)
Supplement: Supplementary file 1 — Appendix S1: [file BJSO-65-0-s001.docx]

**Appendix 1**

*Additional robustness analyses for Study 1*

As part of model diagnostics, we conducted posterior predictive checks to assess the adequacy of the Gaussian specification. These checks suggested that the bounded nature of the outcome variable limited the model’s ability to fully capture the observed distribution (Figure A1). To address this concern, we conducted a robustness analysis in which contact willingness was dichotomized based on the median value, with scores above the median coded as 1 and scores below the median coded as 0. The same multilevel model structure was estimated for this binary outcome using Bayesian logistic regression Table A1. Results were substantively consistent with those obtained for the continuous outcome. In particular, intergroup friendship at the individual level was strongly associated with higher odds of reporting high contact willingness (OR = 4.42, 95% CI [3.23, 6.22]), and the interaction between individual-level intergroup friendship and pre-war contact opportunity was again positive and credible (OR = 1.42, 95% CI [1.05, 1.94]). As in the continuous model, the corresponding interaction at the between-municipality level was not credibly different from zero (OR = 1.18, 95% CI [0.43, 3.34]). Additional robustness analyses showed that the results remained substantively unchanged when using adversary share in 2013 rather than the change in adversary share (Table A2), and when estimating models without control variables (Table A3). Results were also substantively consistent when the model was estimated using Maximum Likelihood Estimation (via the lme4 package) (Table A4).

**
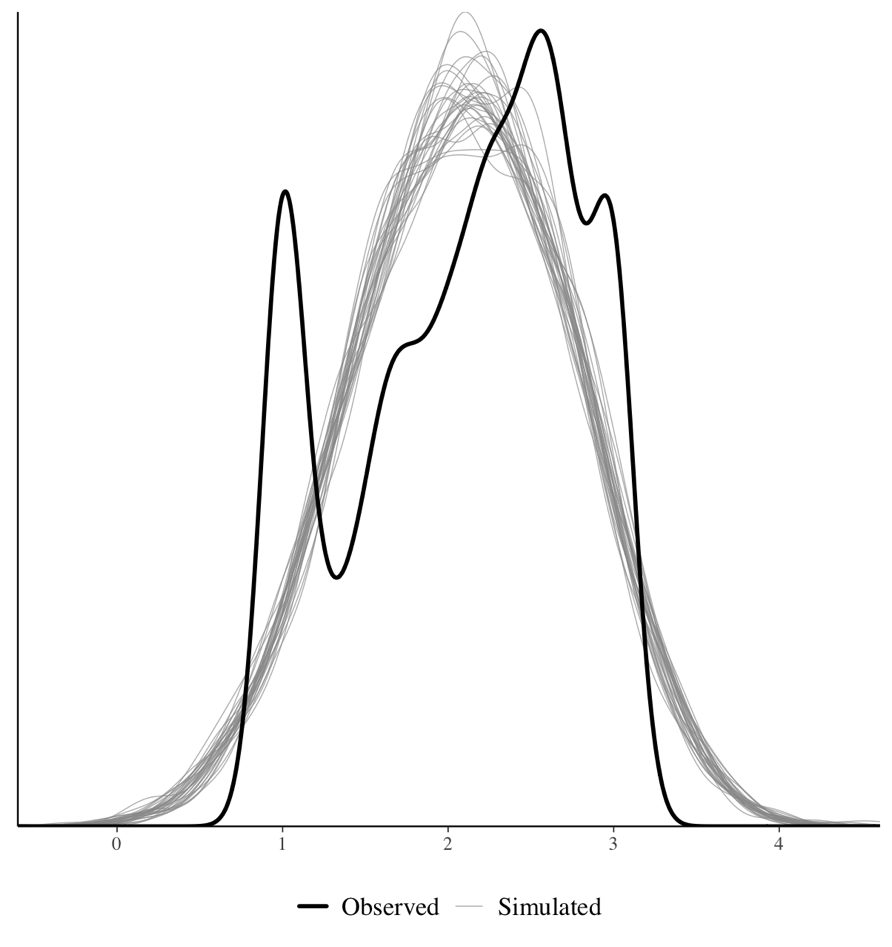
Figure A1**
Posterior Predictive Check for the Bayesian Multilevel Model of Contact Willingness with Adversary Group (Study 1)

**Table A1**

*Bayesian Multilevel Logistic Regression Predicting High Contact Willingness with the Adversary Group (Study 1)*

| Term | OR | | 95% CI |
| --- | --- | --- | --- |
| Intercept | 0.06 | | [0.02, 0.16] |
| Within level friendship | **4.42** | | **[3.23, 6.22]** |
| Between level friendship | **123.96** | | **[24.49, 664.80]** |
| Pre-war contact opportunity | 1.05 | | [0.57, 1.92] |
| Within level friendship × Pre-war contact opportunity | **1.42** | | **[1.05, 1.94]** |
| Between level friendship × Pre-war contact opportunity | 1.18 | | [0.43, 3.34] |
| Age | 1 | | [0.99, 1.01] |
| Sex | 0.84 | | [0.66, 1.09] |
| Ethnic group | **1.7** | | **[1.04, 2.78]** |
| War Experience | 1.13 | | [0.87, 1.46] |
| Type of settlement | 0.9 | | [0.70, 1.16] |
| Adversary share increase | **1.33** | | **[1.11, 1.60]** |
| Pre-war municipal educational attainment | 0.73 | | [0.52, 1.01] |
| Sample size | | 1967 | |
| Number of municipalities | | 105 | |

**Table A2**

*Bayesian Multilevel Linear Regression Predicting High Contact Willingness with the Adversary Group (Study 1)*

| Term | Estimate | | 95% CI |
| --- | --- | --- | --- |
| Intercept | 1.45 | | [1.23, 1.66] |
| Within level friendship | **0.38** | | **[0.31, 0.45]** |
| Between level friendship | **1.10** | | **[0.76, 1.46]** |
| Pre-war contact opportunity | −0.10 | | [−0.22, 0.01] |
| Within level friendship × Pre-war contact opportunity | **0.09** | | **[0.03, 0.16]** |
| Between level friendship × Pre-war contact opportunity | 0.06 | | [−0.13, 0.25] |
| Age | −0.00 | | [−0.00, −0.00] |
| Sex | −0.02 | | [−0.07, 0.02] |
| Ethnic group | **0.12** | | **[0.03, 0.22]** |
| War Experience | 0.02 | | [−0.03, 0.07] |
| Type of settlement | 0.01 | | [−0.04, 0.06] |
| Adversary share in 2013 | **0.14** | | **[0.08, 0.19]** |
| Pre-war municipal educational attainment | −0.03 | | [−0.11, 0.05] |
| Sample size | | 1967 | |
| Number of municipalities | | 105 | |

**Table A3**

*Bayesian Multilevel Linear Regression Predicting High Contact Willingness with the Adversary Group (Study 1)*

| Term | Estimate | | 95% CI |
| --- | --- | --- | --- |
| Intercept | 1.39 | | [1.20, 1.58] |
| Within level friendship | **0.40** | | **[0.33, 0.46]** |
| Between level friendship | **1.21** | | **[0.88, 1.54]** |
| Pre-war contact opportunity | −0.03 | | [−0.15, 0.08] |
| Within level friendship × Pre-war contact opportunity | **0.12** | | **[0.05, 0.18]** |
| Between level friendship × Pre-war contact opportunity | 0.15 | | [−0.03, 0.33] |
| Sample size | | 1967 | |
| Number of municipalities | | 105 | |

**Table A4**

*Maximum-likelihood* *Multilevel Linear Regression Predicting High Contact Willingness with the Adversary Group (Study 1)*

| Term | Estimate | | SE | *t* | | *p* |
| --- | --- | --- | --- | --- | --- | --- |
| Intercept | 1.44 | | 0.1 | 13.77 | | < .001 |
| Within level friendship | **0.38** | | **0.03** | **11.15** | | **< .001** |
| Between level friendship | **1.11** | | **0.17** | **6.51** | | **< .001** |
| Pre-war contact opportunity | 0 | | 0.06 | 0.08 | | .934 |
| Within level friendship × Pre-war contact opportunity | **0.09** | | **0.03** | **2.79** | | **.006** |
| Between level friendship × Pre-war contact opportunity | 0.06 | | 0.1 | 0.61 | | .54 |
| Age | **−0.00** | | **0.00** | **−2.60** | | **.009** |
| Sex | −0.03 | | 0.02 | −1.08 | | .279 |
| Ethnic group | 0.13 | | 0.05 | 2.77 | | .006 |
| War Experience | 0.02 | | 0.02 | 0.73 | | .465 |
| Type of settlement | 0.01 | | 0.02 | 0.44 | | .662 |
| Adversary share increase | **0.09** | | **0.02** | **5.03** | | **< .001** |
| Pre-war municipal educational attainment | −0.03 | | 0.04 | −0.69 | | .494 |
| Sample size | | 1967 | | |  |  |
| Number of municipalities | | 105 | | |  |  |

**Appendix 2**

*Bayesian Multilevel Logistic Regression Predicting High Contact Willingness with the Adversary Group (Study 2 – Objective pre-war contact opportunity)*

| Term | OR | 95% CI |
| --- | --- | --- |
| Intercept | 0 | [0.00, 0.07] |
| Within level positive intergroup contact | **2.61** | **[1.87, 3.84]** |
| Between level positive intergroup contact | **3.48** | **[1.75, 7.54]** |
| Objective pre-war contact opportunity | 0.07 | [0.00, 1.01] |
| Within level positive intergroup contact × Objective pre-war contact opportunity | 1.18 | [0.82, 1.74] |
| Between level positive intergroup contact × Objective pre-war contact opportunity | **1.95** | **[1.04, 3.95]** |
| Age | 1.02 | [0.97, 1.07] |
| Sex | 0.72 | [0.36, 1.44] |
| Ethnic group | 1.71 | [0.56, 5.23] |
| War Experience | 1.13 | [0.56, 2.33] |
| Adversary share increase | 1.11 | [0.66, 1.86] |
| Pre-war municipal educational attainment | 0.8 | [0.45, 1.39] |
| Sample size | 330 | |
| Number of municipalities | 59 | |

**Appendix 3**

*Moderating effect of objective pre-war contact opportunity on the relationship between positive post-war intergroup contact and contact willingness with the adversary group (Study 2)*


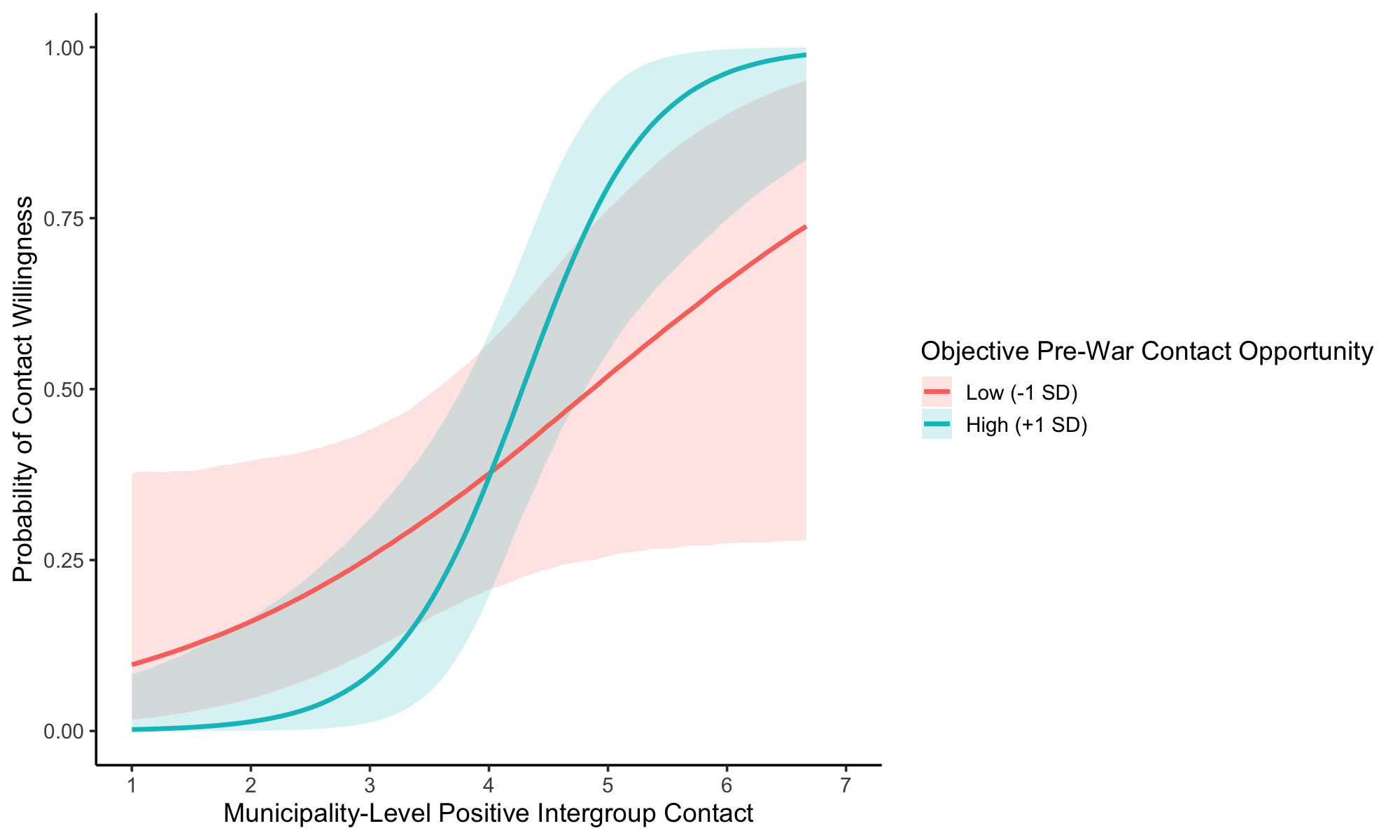


Note. Lines show predicted probabilities at two levels of **objective pre-war contact opportunity** (Low = -1 SD, High = +1 SD). Shaded areas represent 95% credible intervals. Analyses are based on 330 respondents (Level 1) nested within 59 municipalities (Level 2).

**Appendix 4.** *Additional robustness analyses for Study 2 (Objective pre-war contact opportunity)*

To evaluate the fit of the continuous Gaussian multilevel model, we conducted posterior predictive checks using Mplus. In Mplus, cross-level interactions are implemented via **random coefficient prediction**, whereby a level-2 predictor is specified to predict the random slope of a level-1 effect. Posterior predictive p-values (PPP) are not reported for models that include random slopes. Accordingly, to obtain a quantitative posterior predictive fit index, we estimated an otherwise identical auxiliary model excluding the random slope for within-municipality positive intergroup contact. Because the cross-level interaction between pre-war contact opportunity and within-municipality contact is defined through this random slope, its exclusion necessarily entailed omission of this interaction in the auxiliary model. Moderation at the between-municipality level, operationalized as the interaction between municipality-level positive contact and pre-war contact opportunity, remained modeled.

The auxiliary model yielded a posterior predictive p-value of 0.48, with a 95% confidence interval for the difference between the observed and replicated chi-square statistics spanning zero (−13.17, 14.83), indicating acceptable global model fit. In addition, visual posterior predictive checks were conducted in R using the full model reported in the main text. These diagnostics showed correspondence between observed and replicated outcome distributions, with no systematic departures from Gaussian assumptions (Figure B1). Taken together, both the quantitative and visual posterior predictive assessments suggest that the continuous Gaussian specification provided an acceptable representation of the data.

To assess robustness of the findings analysis that followed preregistration where adversary contact williwness was treated as dictimized, we conducted a sensitivity analysis excluding respondents within ±0.10 SD of the median willingness score (Coste & Pouchot, 2003). In this restricted sample (N = 300; 58 municipalities), the previously observed interaction was no longer credibly different from zero (OR = 1.69, 95% CI [0.88, 3.62]; Table B21). This finding suggests that, unlike Study 1 conducted approximately 10 years after the war, Study 2 conducted 30 years after the war does not provide evidence for moderation of within-municipality level contact effect by pre-war contact opportunity. In addition to differences in temporal distance from the conflict, the two studies also differed in how post-war contact was operationalized, which could plausibly contribute to the inconsistency in results. To address this possibility, we re-estimated the models in Study 2 using an intergroup friendship measure rather than contact frequency, approximating the operationalization employed in Study 1. Although the measures were not identical, with Study 1 capturing friendship with members of any outgroup and Study 2 indexing the number of friends from the adversary group, this alternative specification likewise yielded no evidence of moderation (see Table B2).

As described in the main text, Study 2 draws exclusively on participants assigned to the control condition of a larger experimental study comprising approximately 7,200 respondents. Consequently, the final analytic sample consisted of 330 respondents nested within 59 municipalities, which is substantially smaller than the Study 1 sample of 1,967 respondents from 105 municipalities. This difference in sample size may indicate that Study 2 was underpowered to detect moderation effects, offering a plausible alternative explanation for the absence of a significant interaction between pre-war contact opportunity and intergroup friendship.

One key exclusion criterion involved removing respondents who lived in a different municipality prior to the war, as pre-war contact opportunity was measured at the municipality level. Because the questionnaire did not include a direct item assessing whether respondents resided in the same municipality before the war, we used place of birth as a proxy and retained only participants who currently lived in the same municipality in which they were born. This restriction resulted in an analytic sample of 330 respondents. However, the questionnaire also included an item asking whether respondents had moved as a result of the war, which could serve as an alternative proxy for pre-war residential stability. We therefore conducted an additional analysis using this item as the filtering criterion. This approach yielded a larger sample of 647 respondents nested within 95 municipalities. Results from this analysis were substantively identical to those reported in the main findings: pre-war contact opportunity did not emerge as a significant moderator (Table B3). Despite the increased sample size, statistical power remained limited.

To further increase statistical power, we estimated the same model using participants from both the control and experimental conditions, yielding a total sample of 1,831 respondents nested within 99 municipalities. To account for the intervention, we included experimental condition as a set of four dummy variables, with the control group serving as the reference category. With this specification, the analytic sample size and clustering structure were comparable to those in Study 1. Consistent with the main analyses, neither within-level nor between-level positive intergroup contact was credibly moderated by objective pre-war contact opportunity when the outcome was treated as continuous. In the dichotomized specification, however, the interaction between between-level contact and pre-war contact opportunity was supported (OR = 1.26, 95% CI [1.00, 1.59]), with the credible interval narrowly including the null value (Table B4).

In addition to analyses using the objective indicator of pre-war contact opportunity, we conducted supplementary analyses using a perceived measure of pre-war contact opportunity. We included this measure for two reasons. First, because objective pre-war contact opportunity was linked to respondents’ pre-war municipality, analyses based on this indicator required substantial sample restriction due to post-war displacement and limited information on residential continuity. Second, prior research suggests that perceived diversity may shape intergroup relations over and above objective diversity and may in some cases mediate its effects (Ramos et al., 2016). Thus, although our main analyses focus on the objective contextual indicator, the perceived measure provides a useful supplementary test of whether respondents’ subjective representations of the pre-war intergroup context show a similar moderating pattern.

Perceived pre-war contact opportunity was measured with the item: *“Before the war, approximately what percentage of people living in your area were Serbs/Bosniaks?”* Responses were given as percentages and converted into proportions by dividing by 100, such that higher values reflected greater perceived pre-war contact opportunity with the adversary group. As with the objective indicator, the relevant adversary group was defined relative to respondents’ own ethnicity (i.e., perceived proportion of Serbs for Bosniak respondents and perceived proportion of Bosniaks for Serb respondents). Using this measure, we tested moderation for both continuous and dichotomized outcomes (Table B5 for descriptives). Consistent with the main Study 2 analyses, moderation did not emerge in the continuous models, whereas some evidence of moderation appeared only in the dichotomized models (Table B6; see also Figure B2 and Figure B3 for visualizations). To evaluate whether the absence of moderation in the continuous models could plausibly be attributed to limited power, we conducted a simulation-based power analysis anchored in the interaction observed in Study 1 (B = 0.089, 95% CI [0.03, 0.15]). After standardizing this effect and rescaling it (β = 0.065) to the metric of Study 2, the corresponding target interaction was approximately B = 0.13. Monte Carlo simulations preserving the observed multilevel structure and variance components of Study 2 indicated approximately 0.76 power to detect an interaction of this magnitude or larger. Although slightly below the conventional 0.80 benchmark, this level of power suggests that the Study 2 design was reasonably sensitive to effects of this size.

**Figure B1**

Posterior Predictive Check for the Bayesian Multilevel Model of Contact Willingness with Adversary Group (Study 2)

**
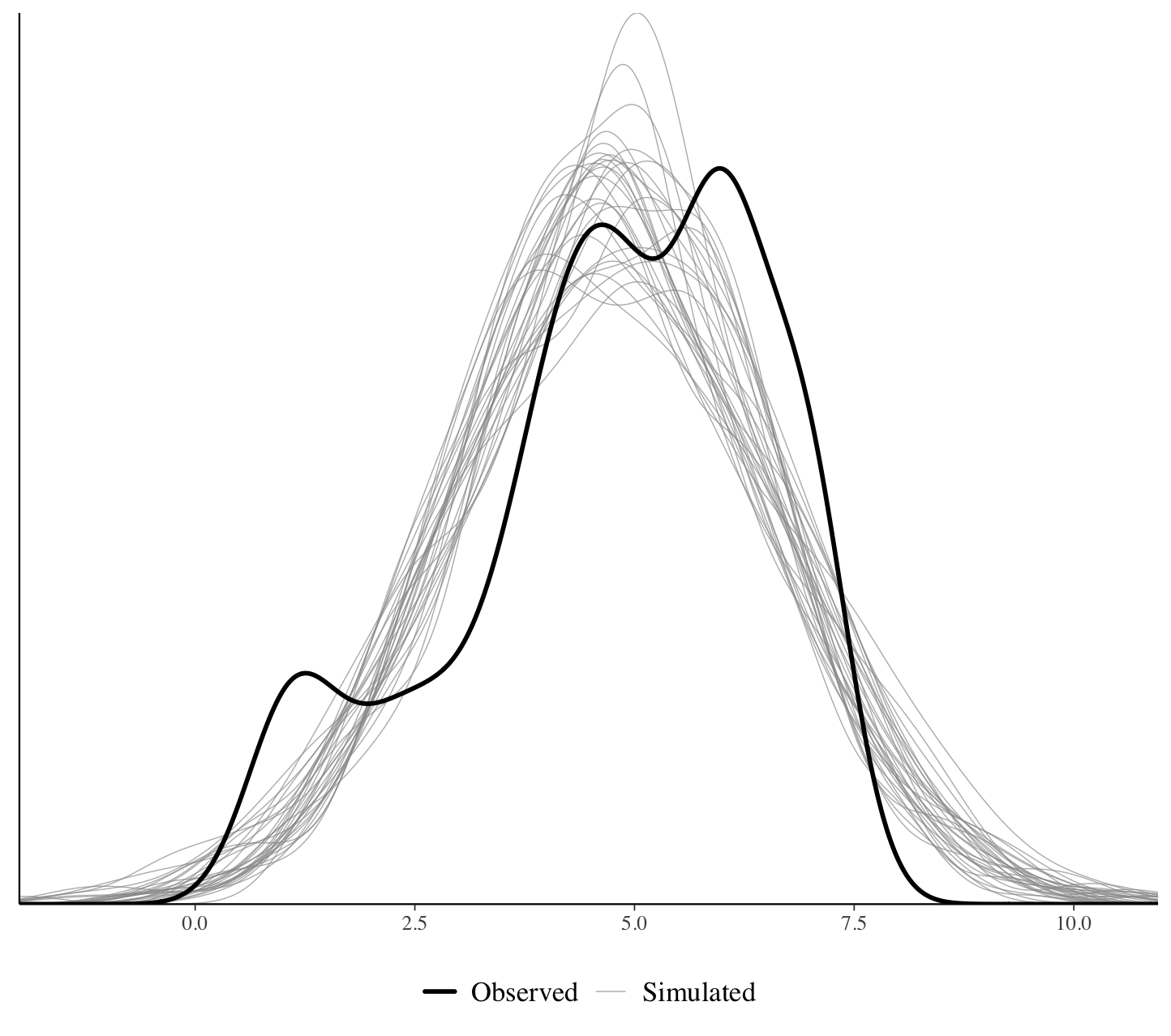
**

**Table B1**

*Bayesian Multilevel Logistic Regression Predicting High Contact Willingness with the Adversary Group (Study 2 – Objective pre-war contact opportunity)*

| Term | OR | 95% CI |
| --- | --- | --- |
| Intercept | 0.00 | [0.00, 0.03] |
| Within level positive intergroup contact | **2.57** | **[1.79, 3.86]** |
| Between level positive intergroup contact | **4.68** | **[2.32, 10.77]** |
| Objective pre-war contact opportunity | 0.1 | [0.00, 1.67] |
| Within level positive intergroup contact × Objective pre-war contact opportunity | 1.21 | [0.83, 1.84] |
| Between level positive intergroup contact × Objective pre-war contact opportunity | 1.69 | [0.88, 3.62] |
| Age | 1.02 | [0.97, 1.08] |
| Sex | 0.63 | [0.29, 1.30] |
| Ethnic group | 2.31 | [0.72, 7.54] |
| War Experience | 1.10 | [0.50, 2.40] |
| Adversary share increase | 1.17 | [0.68, 2.01] |
| Pre-war municipal educational attainment | 0.80 | [0.47, 1.34] |
| Sample size | 300 | |
| Number of municipalities | 58 | |

**Table B2**

*Bayesian Multilevel Regression Predicting High Contact Willingness with the Adversary Group (Study 2 – Objective pre-war contact opportunity)*

|  | Continuous outcome | |  | Dichotomized outcome | |
| --- | --- | --- | --- | --- | --- |
| Term | Estimate | 95% CI |  | OR | 95% CI |
| Intercept | -8.17 | [-12.82, -3.87] |  | 0 | [0.00, 0.06] |
| Within level intergroup friendship | **1.03** | **[0.69, 1.40]** |  | **2.68** | **[1.90, 4.20]** |
| Between level intergroup friendship | **2.07** | **[1.37, 2.94]** |  | **5.02** | **[2.78, 9.96]** |
| Objective pre-war contact opportunity | -2.11 | [-5.01, 0.44] |  | 0.22 | [0.02, 1.89] |
| Within level intergroup friendship × Objective pre-war contact opportunity | 0.08 | [-0.23, 0.40] |  | 1.12 | [0.81, 1.55] |
| Between level intergroup friendship × Objective pre-war contact opportunity | 0.39 | [-0.21, 1.06] |  | 1.34 | [0.82, 2.29] |
| Age | 0 | [-0.07, 0.06] |  | 1 | [0.94, 1.05] |
| Sex | -0.8 | [-1.68, 0.02] |  | 0.56 | [0.27, 1.15] |
| Ethnic group | 0.92 | [-0.26, 2.13] |  | 1.48 | [0.51, 4.11] |
| War Experience | -0.02 | [-0.86, 0.84] |  | 0.92 | [0.44, 1.93] |
| Adversary share increase | 0.04 | [-0.49, 0.58] |  | 1.04 | [0.63, 1.67] |
| Pre-war municipal educational attainment | -0.33 | [-0.88, 0.17] |  | 0.74 | [0.43, 1.22] |
| Sample size | 328 | | | | |
| Number of municipalities | 59 | | | | |

**Table B3**

*Bayesian Multilevel Logistic Regression Predicting High Contact Willingness with the Adversary Group (Study 2 – Objective pre-war contact opportunity)*

| Term | Estimate | 95% CI |
| --- | --- | --- |
| Intercept | 2.09 | [0.97, 3.23] |
| Within level positive intergroup contact | **0.51** | **[0.41, 0.60]** |
| Between level positive intergroup contact | **0.51** | **[0.34, 0.68]** |
| Objective pre-war contact opportunity | −0.09 | [−0.65, 0.48] |
| Within level positive intergroup contact × Objective pre-war contact opportunity | −0.07 | [−0.17, 0.03] |
| Between level positive intergroup contact × Objective pre-war contact opportunity | 0.04 | [−0.10, 0.17] |
| Age | 0.01 | [−0.00, 0.03] |
| Sex | −0.17 | [−0.38, 0.05] |
| Ethnic group | **0.42** | **[0.12, 0.73]** |
| War Experience | −0.19 | [−0.42, 0.03] |
| Adversary share increase | **0.15** | **[0.02, 0.28]** |
| Pre-war municipal educational attainment | 0.01 | [−0.14, 0.16] |
| Sample size | 647 | |
| Number of municipalities | 95 | |

**Table B4**

*Bayesian Multilevel Logistic Regression Predicting High Contact Willingness with the Adversary Group (Study 2 – Objective pre-war contact opportunity)*

|  | Continuous outcome | |  | Dichotomized outcome | |
| --- | --- | --- | --- | --- | --- |
| Term | Estimate | 95% CI |  | OR | 95% CI |
| Intercept | 2.57 | [1.75, 3.40] |  | 0.02 | [0.00, 0.09] |
| Within level positive intergroup contact | **0.48** | **[0.42, 0.53]** |  | **2.03** | **[1.83, 2.29]** |
| Between level positive intergroup contact | **0.54** | **[0.39, 0.70]** |  | **2.29** | **[1.68, 3.13]** |
| Objective pre-war contact opportunity | −0.29 | [−0.75, 0.17] |  | 0.35 | [0.13, 0.90] |
| Within level positive intergroup contact × Objective pre-war contact opportunity | −0.03 | [−0.08, 0.02] |  | 1.03 | [0.93, 1.15] |
| Between level positive intergroup contact × Objective pre-war contact opportunity | 0.07 | [−0.04, 0.18] |  | **1.26** | **[1.00, 1.59]** |
| Age | 0.00 | [−0.01, 0.01] |  | 1 | [0.99, 1.02] |
| Sex | **−0.14** | **[−0.26, −0.02]** |  | 0.77 | [0.61, 0.98] |
| Ethnic group | **0.20** | **[0.00, 0.39]** |  | 1.14 | [0.79, 1.63] |
| War Experience | **−0.20** | **[−0.32, −0.07]** |  | 0.76 | [0.59, 0.97] |
| Adversary share increase | **0.12** | **[0.05, 0.20]** |  | **1.27** | **[1.09, 1.49]** |
| Pre-war municipal educational attainment | 0.03 | [−0.08, 0.14] |  | 1.08 | [0.89, 1.32] |
| Experimental condition 1 | **0.19** | **[0.01, 0.36]** |  | 1.22 | [0.87, 1.71] |
| Experimental condition 2 | 0.18 | [−0.00, 0.36] |  | 1.25 | [0.89, 1.76] |
| Experimental condition 3 | **0.18** | **[0.01, 0.35]** |  | 1.37 | [0.97, 1.92] |
| Experimental condition 4 | **0.23** | **[0.05, 0.41]** |  | 1.4 | [0.99, 2.00] |
| Sample size | 1831 | | | | |
| Number of municipalities | 99 | | | | |

**Table B5**

*Descriptive Statistics and Bivariate Correlations (Study 2 -* ***Perceived pre-war contact opportunity*** *)*

| Variable | M | SD | 1 | 2 | 3 | 4 | 5 | 6 | 7 | 8 |
| --- | --- | --- | --- | --- | --- | --- | --- | --- | --- | --- |
| 1. Positive intergroup contact | 4.08 | 1.61 |  |  |  |  |  |  |  |  |
| 2. **Perceived pre-war contact opportunity** | 0.23 | 0.25 | .25** |  |  |  |  |  |  |  |
| 3. Contact willingness with adversary group | 4.83 | 1.63 | .55** | .08** |  |  |  |  |  |  |
| 4. Age | 55.56 | 6.72 | -.04 | -.05 | -.00 |  |  |  |  |  |
| 5. Sex | 0.45 | 0.5 | .03 | -.10** | -.02 | .08** |  |  |  |  |
| 6. Ethnic group | 0.45 | 0.5 | .02 | 0.05 | .11** | -.03 | 0.04 |  |  |  |
| 7. War experience | 0.46 | 0.5 | -.00 | 0.01 | -.05 | .16** | .15** | .15** |  |  |
| 8. Adversary share increase | -0.10 | 0.16 | .08** | .12** | .13** | 0.03 | 0.02 | .09** | .08** |  |
| 9. Pre-war municipal educational attainment | 0.06 | 0.04 | .08** | .08* | .05 | -.00 | 0.01 | 0.04 | 0.03 | -.16** |

*Note.* *M* and *SD* are used to represent mean and standard deviation, respectively. * indicates *p* < .05. ** indicates *p* < .01.

**Table B6**

*Bayesian Multilevel Linear Regression Predicting High Contact Willingness with the Adversary Group (Study 2 - Perceived pre-war contact opportunity)*

|  | Continuous outcome | |  | Dichotomized outcome | |
| --- | --- | --- | --- | --- | --- |
| Term | Estimate | 95% CI |  | OR | 95% CI |
| Intercept | 2.26 | [1.33, 3.21] |  | 0 | [0.00, 0.04] |
| Within level positive intergroup contact | **0.54** | **[0.46, 0.61]** |  | **2.71** | **[2.25, 3.37]** |
| Between level positive intergroup contact | **0.61** | **[0.44, 0.77]** |  | **3.15** | **[2.16, 4.67]** |
| Within level perceived pre-war contact opportunity | −0.22 | [−0.78, 0.33] |  | 0.19 | [0.04, 0.81] |
| Between level perceived pre-war contact opportunity | 0.4 | [−0.74, 1.52] |  | 0.47 | [0.04, 5.61] |
| Within level positive intergroup contact × Within level perceived pre-war contact opportunity | 0.04 | [−0.03, 0.11] |  | **1.38** | **[1.13, 1.70]** |
| Between level positive intergroup contact × Within level perceived pre-war contact opportunity | 0.05 | [−0.08, 0.17] |  | **1.44** | **[1.04, 2.02]** |
| Within level positive intergroup contact × Between level perceived pre-war contact opportunity | −0.07 | [−0.21, 0.06] |  | 1.07 | [0.80, 1.42] |
| Between level positive intergroup contact × Between level perceived pre-war contact opportunity | −0.16 | [−0.41, 0.10] |  | 1.03 | [0.59, 1.77] |
| Age | 0.00 | [−0.01, 0.01] |  | 1.01 | [0.98, 1.04] |
| Sex | −0.12 | [−0.28, 0.04] |  | 0.69 | [0.49, 0.97] |
| Ethnic group | **0.29** | **[0.08, 0.50]** |  | **1.66** | **[1.05, 2.63]** |
| War Experience | −0.21 | [−0.37, −0.05] |  | 0.63 | [0.45, 0.91] |
| Adversary share increase | **0.15** | **[0.06, 0.25]** |  | **1.28** | **[1.04, 1.60]** |
| Pre-war municipal educational attainment | 0.06 | [−0.06, 0.17] |  | 1.06 | [0.83, 1.34] |
| Intercept | 2.26 | [1.33, 3.21] |  | 0 | [0.00, 0.04] |
| Sample size | 1095 | | | | |
| Number of municipalities | 98 | | | | |

**Figure B2**

*Moderating effect of perceived pre-war contact opportunity on the relationship between positive post-war intergroup contact (Within-Municipality) and contact willingness with the adversary group (Study 2)*

**
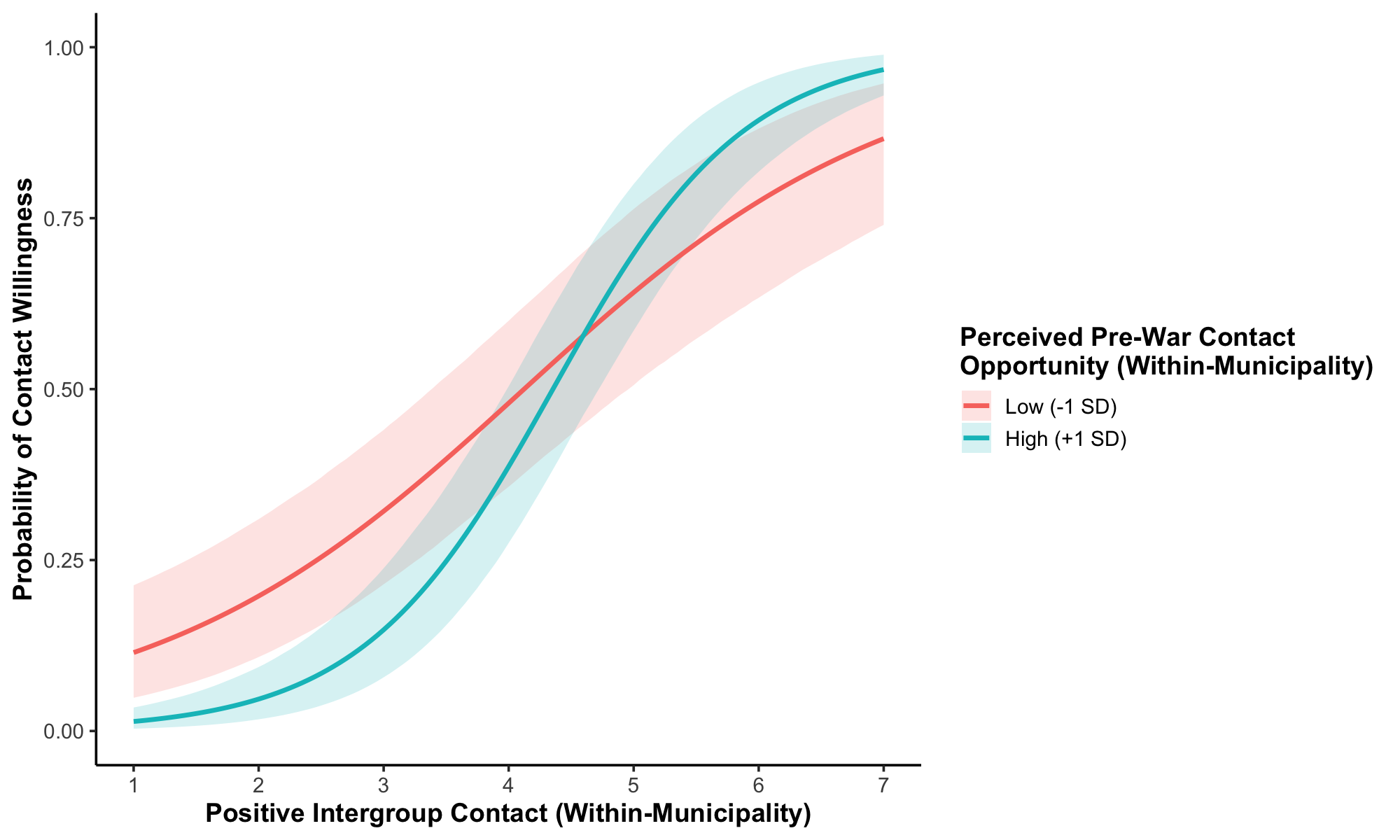
**

Note. Lines show predicted probabilities at two levels of **perceived pre-war contact opportunity** (Low = -1SD, High = +1SD). The x-axis shows positive intergroup contact on its raw 1–7 scale; predictions are generated by converting this value into the within-municipality deviation from the municipality contact mean, holding the municipality mean fixed at its sample average. Shaded areas represent 95% credible intervals. Analyses are based on 1095 respondents (Level 1) nested within 98 municipalities (Level 2).

**Figure B3**

*Moderating effect of perceived pre-war contact opportunity on the relationship between positive post-war intergroup contact (Between-Municipality) and contact willingness with the adversary group (Study 2)*

**
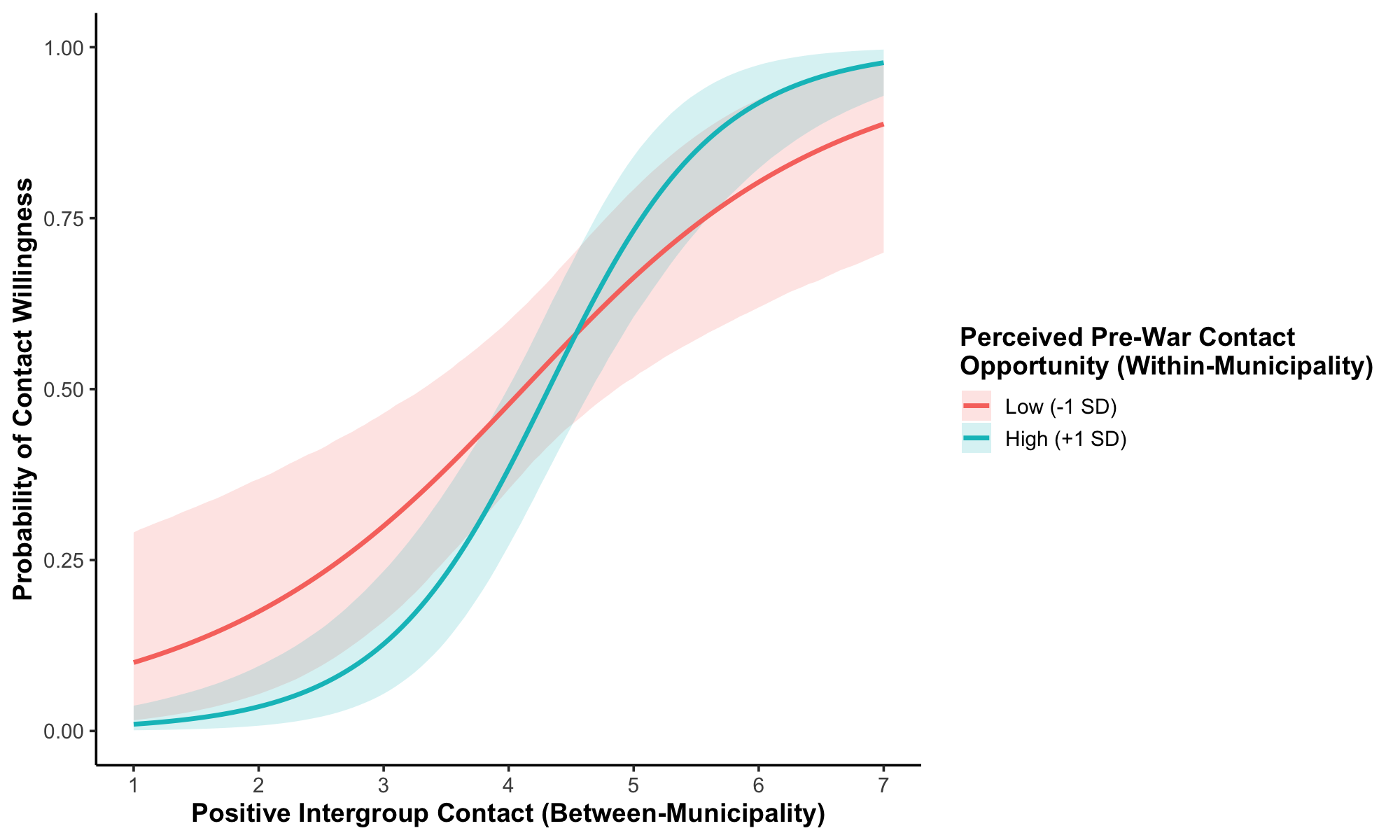
**

Note. Lines show predicted probabilities at two levels of **perceived pre-war contact opportunity** (Low = -1SD, High = +1SD). The x-axis displays **between-municipality** positive intergroup contact on its raw 1–7 scale. Shaded areas represent 95% credible intervals. Analyses are based on 1095 respondents (Level 1) nested within 98 municipalities (Level 2).

Coste, J., & Pouchot, J. (2003). A grey zone for quantitative diagnostic and screening tests. *International Journal of Epidemiology*, *32*(2), 304–313. https://doi.org/10.1093/ije/dyg054

Kende, J., Jacobs, D., Green, E. G. T., Tropp, L. R., Huo, Y. J., Dovidio, J. F., Jiménez, T. R., Schildkraut, D. J., & Klein, O. (2024). Integration policies shape ethnic-racial majorities’ threat reactions to increasing diversity. *Science Advances*, *10*(22), eadk8556. https://doi.org/10.1126/sciadv.adk8556

Mathieu, J. E., Aguinis, H., Culpepper, S. A., & Chen, G. (2012). Understanding and estimating the power to detect cross-level interaction effects in multilevel modeling. *Journal of Applied Psychology*, *97*(5), 951–966. https://doi.org/10.1037/a0028380

Ramos, M. R., Hewstone, M., Barreto, M., & Branscombe, N. R. (2016). The opportunities and challenges of diversity: Explaining its impact on individuals and groups. *European Journal of Social Psychology*, *46*(7), 793–806. https://doi.org/10.1002/ejsp.2261
